# 免疫检查点抑制剂相关性重症肌无力：从诊断到治疗

**DOI:** 10.3779/j.issn.1009-3419.2020.102.25

**Published:** 2020-08-20

**Authors:** 舒洋 姚, 小雪 李, 靖颖 农, 毅 张

**Affiliations:** 100053 北京，首都医科大学宣武医院胸科 首都医科大学肺癌诊疗中心 Department of Thoracic Surgery, Xuanwu Hospital, Diagnostic and Treatment Centers of Lung Cancer, Capital Medical University, Beijing 100053, China

**Keywords:** 免疫检查点抑制剂, 重症肌无力, 外周神经病变, 免疫治疗不良反应, Immune checkpoint inhibitor, Myasthenia gravis, Peripheral neuropathy, Immune-related adverse events

## Abstract

已经证实免疫检查点抑制剂（immune checkpoint inhibitor, ICI）使多种肿瘤的治疗有了翻天覆地的变化。尽管治疗的有效性让人欣慰，但是这些治疗也造成了多样化的免疫治疗相关的不良反应（immune-related adverse events, irAEs）。重症肌无力（myasthenia gravis, MG）就是一种罕见且危及生命的irAE，通常在ICI治疗后急性发病并迅速进展。早期诊断和积极的治疗非常重要。在此，我们对近几年的相关文献进行了复习和整理，针对ICI-MG诊断和治疗相关的常见问题提供参考意见。

## Abstract (en)

Immune checkpoint inhibitor (ICI) has been proven to be a major breakthrough in the treatment of various tumor types. Despite the favorable results in terms of oncological outcomes, these treatments have been associated with a variety of immune-related adverse events (irAEs). Myasthenia gravis (MG) is one of rare but life-threatening irAEs, with acute onset and rapid progression after ICI initiation. Early diagnosis and active treatment are crucial. Herein, we review recent literatures to provide guidance to frequently asked questions concerning the diagnosis and management of ICI-MG.

目前，我们已经进入了免疫检查点抑制剂（immune checkpoint inhibitor, ICI）的时代，ICIs明显延长了多种实体瘤患者的生存时间。这些药物可以单独使用，也可以与其他的免疫治疗药物或者与化疗联合应用。

作为医生，我们一方面惊喜于这类药物的疗效，一方面也受到大量免疫相关不良反应（immune-related adverse events, irAEs）的困扰。虽然大部分患者的irAEs是不需要特别处理且不需要中断治疗的Ⅰ级-Ⅱ级反应，最常见的部位是皮肤（44%, 95%CI: 38%-49.5%）、消化道（35%, 95%CI: 29%-41%）^[1]^，但是随着上市的药物越来越多，适应证越来越广，而且ICI已经从晚期肿瘤治疗领域推进到围手术期，未来将有越来越多的早期肿瘤患者接受ICI治疗，之前少见甚至是罕见的严重irAEs的数量会相应增多，这就对肿瘤科医生提出了很高的要求。

一篇包含了9, 208例患者、59项有关ICI临床试验在内的研究^[2]^发现，细胞毒性T淋巴细胞相关蛋白4（cytotoxic T-lymphocyte antigen 4, CTLA-4）抑制剂相关的神经系统irAEs发生率为6.1%，程序性死亡受体-1（programmed cell death-1, PD-1）/程序性死亡受体配体1（programmed cell death ligand 1, PD-L1）抑制剂相关的神经系统irAEs发生率为3.8%，而CTLA-4抑制剂与PD-1抑制剂联合应用的发生率为12%，其中大部分为1级-2级，以头疼等非特异性症状为主；而PD-1/PD-L1抑制剂所致3级-5级严重irAEs发生率为0.4%。还有其他文章也得到类似的结果，3级-4级神经系统irAEs发生率小于1%^[3, 4]^。虽然神经系统的irAEs发生率远低于其他器官的irAEs，但不能排除有医生误诊或者过低的上报，而低估了其真实的发病率。

重症肌无力（myasthenia gravis, MG）是一种神经肌肉接头传递功能障碍所引起的疾病，也是神经系统irAEs的一种。我们最近救治过1例相关患者，病情变化非常迅速，治疗及时而积极，患者完全恢复。然而，这一不良反应的患者如果不能早期明确诊断给予积极有效的治疗，病死率非常高。在此，对ICI相关MG的诊治特点，我们进行了综述。

## 1 ICI-MG发病机制

irAEs的发生与免疫系统的过度活化、异位抗原的表达、抗体依赖性反应、细胞因子的增多、遗传倾向等因素有关。由于irAEs患者组织活检结果非常多样，反而影响了我们对irAEs发病机制的理解：不仅患者不同组织表现出不同的发病机制，而且出现同一种irAE的2例患者，其发病机制也不相同。

有关ICI-MG发病机制的研究非常有限。1例使用纳武单抗治疗后发展为MG/肌炎/心肌炎的患者在治疗前后接受过外周血粒细胞的基因表达分析，发现CD8^+^ T细胞增多、细胞溶解活性标志物表达增高；而CD4^+^ T细胞数量减少，T调节细胞活性受到抑制^[5]^。还有1例使用纳武单抗治疗后发展为MG/肌炎的患者，外周血淋巴细胞的CD8/CD4比率为1.4^[6]^。CD4/CD8比例倒置提示抑制性T细胞致敏，说明了ICI引起免疫应答过度激活。肌肉组织活检发现中重度的坏死伴有巨噬细胞和T细胞局灶性聚集浸润，形成假性肉芽肿结构^[7]^。不管这些结果是否与MG发病相关，但确实与ICI治疗有关。

## 2 ICI-MG的诊断

ICI-MG可以是既往MG病情的加重或复发（在ICI治疗前就有明确的MG病史），也可以是ICI治疗后新发的MG（既往没有MG病史，ICI治疗后出现的）；单纯的MG比较少见，更多的是MG合并肌炎和/或心肌炎，而后者更常快速发展成肌无力危象，需要重症监测和机械通气支持。

### 2.1 ICI-MG的好发时间

ICI治疗后，患者首次出现MG症状的中位时间为4周（6 d-16周）^[8]^，大部分出现在ICI治疗2周期后。与其他系统的irAEs相比，MG更常发生在ICI治疗的早期。在ICI治疗开始的3个月-4个月内需要提高警惕。

### 2.2 ICI-MG常见临床表现或体征

ICI-MG的常见临床表现为眼部症状（50%以上的患者有上睑下垂和/或复视）、四肢肌力减退、呼吸困难和吞咽困难。约2/3的患者出现美国重症肌无力基金会（Myasthenia Gravis Foundation of America, MGFA）分级为5级的严重肌力减退，约45%的患者快速进展出现危及生命的呼吸肌肌力减退^[8]^。这些临床表现多为非特异性的，给早期诊断造成了很大挑战：很多患者出现乏力、轻度肌肉疼痛和四肢肌力减退，这与晚期肿瘤的相关表现难以区别，很容易被患者和肿瘤科医生忽视；而且患者在症状轻微时，多隐忍不说，到症状明显的时候才与医生沟通，很容易丧失最佳诊断和治疗的时间窗；肿瘤科医生缺乏神经irAEs的诊疗能力；而神经科医生也会因为这些表现非常不典型，而且对ICI药物不熟悉，而面临诊断和治疗中存在的很多困难。

### 2.3 ICI-MG实验室和其他检查特点

需要对患者进行脑核磁、脑脊液、肌电图、大量的抗体检测以排除其他，如血管性疾病、肿瘤进展（脑转移、脊髓压迫和脑膜转移）、感染性疾病、副肿瘤综合症或中毒、代谢性疾病，完成鉴别诊断。需要胸部CT除外胸腺的病变，心脏超声明确左心室功能。必要时进行骨骼肌和心肌活检明确是否合并有肌炎和心肌炎。值得注意的是，只有59%的患者存在乙酰胆碱能受体（acetylcholine receptor, AchR）阳性^[9]^。当患者合并有肌炎和/或心肌炎时，会有肌酸激酶（creatine kinase, CK）的明显升高，合并心肌炎的患者会出现肌钙蛋白的升高。

### 2.4 ICI-MG与自发性-MG（idiopathic MG, iMG）的不同特点

ICI-MG与iMG有明显的差异：①根据MGFA来判断MG的严重程度，iMG患者4级/5级约占2%-10%^[10, 11]^；而ICI-MG 4级/5级的患者占50%；②从出现MG症状开始进展为4级，iMG缓慢进展，需要2年-3年的时间^[12]^；而ICI-MG患者，中位进展时间为7 d（24 h-60 d）；③与iMG相比，ICI-MG更常合并肌炎和/或心肌炎。有文献^[10]^报道，30%的患者合并肌炎，25%的患者合并心肌炎；而iMG合并心肌炎的患者仅为0.9%^[13]^，iMG更常见的伴随疾病是胸腺瘤；④ICI-MG的AChR阳性率明显低于iMG，而ICI-MG的肌炎相关抗体的阳性率明显高于没有胸腺瘤的iMG；⑤治疗上来说，胆碱受体激动剂更常应用于iMG的治疗，激素可能会加重iMG的症状；而ICI-MG的治疗过程中，激素为主要治疗手段。

## 3 ICI-MG的治疗

虽然只有完成所有相关鉴别诊断才能明确ICI-MG的诊断，但是，肌无力症状的出现与ICI用药时间的密切关系非常有助于肿瘤科医生作出诊断，并迅速开始相关治疗。因为，早期治疗对本病的预后非常重要。

### 3.1 ICI-MG对症治疗

在暂停ICI治疗的基础上，主要有3种治疗方式：对症治疗（抗胆碱酯酶抑制剂：吡啶斯的明）、慢性免疫调节剂（糖皮质激素和免疫抑制剂）和快速免疫调节剂[静脉用丙种球蛋白（intravenous immunoglobulin, IVIG）和血浆置换术（plasmapheresis, PLEX）]。目前，暂停或永久终止ICI治疗并使用皮质类固醇是临床上最常用的治疗方式。抗胆碱酯酶（acetylcholinesterase, AChE）抑制剂（吡啶斯的明）大多用于维持治疗阶段，它单独使用的效果并不理想，除非患者症状轻微而且症状稳定无任何进展。鉴于皮质类固醇药物的诸多副作用，也有推荐吡啶斯的明与类固醇药物的联合应用，以降低类固醇药物的用量^[9]^。

一项MD Anderson医院的回顾性分析^[8]^结果发现把IVIG或者PLEX作为一线治疗的患者预后优于一线只应用激素治疗的患者（MG症状改善率：95% *vs* 63%，*P*=0.011），而且前者起效快。皮质类固醇，常用强的松1 mg/kg，每天1次，能使1/3-1/2的患者出现暂时性症状加重，但最终会有70%-75%的患者达到明显缓解或好转^[14]^。值得注意的是，暂时性症状加重可能导致10%的患者需要机械通气来改善呼吸衰竭。因此，可以对入院患者进行一线皮质类固醇联合IVIG或血浆置换术的治疗，这也是与其他irAEs治疗有明显区别的，因单用皮质类固醇治疗有加重MG危象的风险，在ICI-MG的治疗中，可采用联合治疗的方法。

### 3.2 ICI-MG的预后

总体来说，有19%的患者MG症状完全缓解，55%症状改善，26%症状恶化。有一半的患者出院后仍需要长期的维持治疗，其中84%的患者接受皮质类固醇治疗，32%接受抗胆碱酯酶抑制剂，16%接受IVIG^[8]^。ICI-MG大部分患者的死亡发生在MG首发症状之后的6周（3周-26.5周），绝大多数合并了肌炎和/或心肌炎。CK和/或肌钙蛋白升高的患者病情进展更为迅速（29% *vs* 13%）、死亡率更高（29% *vs* 6%）^[8]^。

### 3.3 ICI-MG的后续ICI治疗

ICI-MG症状好转后，是否能继续应用ICI治疗目前尚无定论，还没有充足的病例数来证明恢复ICI治疗的安全性。MD Anderson的研究者^[8]^总结的65例患者中有6例新发ICI-MG患者在MG症状缓解后继续使用了ICI。其中3例发病时仅有眼部症状和轻度的乏力，其他3例出现更明显的乏力。这6例患者在症状完全缓解之后，一直使用ICI-MG的维持治疗。从MG初发症状到再次应用ICI的时间为7 d-17.75个月。5例患者再次应用了之前的药物，1例患者更换了ICI药物。这些患者未再次发生症状闪烁的情况。其他研究^[15, 16]^也报道了继续使用ICI治疗后并没有再次出现MG症状恶化的病例。

ICI-MG治疗后再次使用ICI治疗之前，要对患者的肿瘤ICI治疗反应、不良反应的严重程度（不良反应分级为1级、2级以及一部分治疗后症状快速消失的3级）、继续MG维持治疗的可能性和当前身体状况进行综合评估；而且，需要与有神经系统irAEs治疗经验的神经科医生保持密切合作。再次使用ICI时，可以使用原来的药物^[17]^，也可以换用其他类型的ICI^[18]^。

## 4 总结

ICI-MG是一种在ICI应用早期出现的危及生命的irAE，具有症状特异性不强、发病急、进展迅速的特点。早期多学科的联合诊断和治疗至关重要。因为发病率低，目前的治疗意见仅仅来源于经验性资料。我们需要更深入的临床前的研究来了解这一irAE的发病机制和发展特点，也期待着能开展多中心临床研究来寻找最理想的治疗方案。
